# First case of MPox in Pakistan: What can we learn from it?

**DOI:** 10.1002/hsr2.1786

**Published:** 2024-01-07

**Authors:** Areeba Fareed, Aariz Hussain, Fatima Faraz, Rima Siblini

**Affiliations:** ^1^ Karachi Medical and Dental College Karachi Pakistan; ^2^ Department of Medicine Rawalpindi Medical University Rawalpindi Pakistan; ^3^ Faculty of Medical Sciences Lebanese University Hadath Beirut Lebanon

**Keywords:** monkeypox, prevention, public health

## Abstract

**Background and Aims:**

This research paper discusses Pakistan's healthcare systems' challenges in managing the monkeypox outbreak in Pakistan. Monkeypox is a zoonotic viral disease caused by the monkeypox virus. The World Health Organization declared it a global public health emergency due to the surge in cases worldwide. Pakistan reported its first verified case in April 2023, necessitating immediate response.

**Body:**

This correspondence emphasizes on early detection, rapid diagnostic testing, and prompt treatment to limit the virus's transmission. Prevention strategies such as vaccination and travel restrictions on suspected cases are crucial for containment. Lessons from controlling the coronavirus disease 2019 pandemic can inform strategies for monkeypox.

**Conclusion:**

The recent outbreak of monkeypox has presented challenges to healthcare systems. Therefore, a coordinated approach involving healthcare providers, government organizations, and the public is crucial in controlling the monkeypox outbreak. Further research is necessary to understand the virus and develop effective interventions for future outbreaks.

Dear Editor,

The global healthcare sector is presently confronting the obstacle of controlling the resurgent coronavirus disease 2019 virus. To add to this challenge, the recent outbreak of the monkeypox virus (MPXV) has further strained the economic and healthcare systems of various countries. Monkeypox is a zoonotic viral disease caused by the MPXV, an orthopoxvirus belonging to the Poxviridae family of viruses. Although MPXV was first identified in 1958, sporadic cases in humans were first reported in the 1970s across several African countries.^1^ The MPXV enters the human body through multiple routes, such as oropharyngeal, nasopharyngeal, or intradermal routes.^2^ The onset of classic monkeypox is marked by a prodrome of fever, fatigue, and lymphadenopathy, followed by facial skin eruptions, predominantly.^2^

The first outbreak of monkeypox outside of Africa was reported in the United States back in 2003, and the first cases of the current multinational outbreak were identified in Europe on May 6, 2022.^3^ The current outbreak has presented atypical symptoms, which is comprised of a high incidence of mucocutaneous lesions, sore throat, rectal pain, penile edema.^2^ Keeping in view the significant surge in the number of monkeypox cases worldwide, the World Health Organization (WHO) proclaimed the monkeypox epidemic a global public health emergency at the end of June 2022. As of January 2022, 87,000 cases of monkeypox have been reported through laboratory testing, resulting in 120 fatalities in 110 member states across all six WHO regions.^4^ On July 15, 2022, the initial occurrence of monkeypox in the WHO South‐East Asia Region was reported from India, in a 35‐year‐old male who had recently traveled to the Middle East.^5^

Since May 2022, a total of 22 suspected samples from all across Pakistan have been referred to the National Institute of Health (NIH). Subsequent polymerase chain reaction (PCR) testing for the MPXV which came out negative.^6^ The first officially verified case of monkeypox in Pakistan was documented on April 21, 2023, in the Islamabad Capital Territory. The virus was detected in a 25‐year‐old Pakistani man who had recently come back from Saudi Arabia.^7^ A fellow passenger who was seated with the infected individual on the flight also manifested symptoms of monkeypox. As a result, both individuals were promptly isolated and hospitalized. In addition, their family members, close contacts, and other passengers who were in close proximity were also isolated and advised strict surveillance in terms of any potential symptoms.^8^ To mitigate any further spread of the virus, the NIH established a multidisciplinary team along with provincial health departments, border health services, and district health authorities. They have all been instructed to maintain vigilant surveillance measures through the use of laboratory diagnostics, contact tracing, prompt identification of suspected cases, and providing medical care while isolating infected individuals to prevent the spread of the virus.

The clinical signs and symptoms of MPXV infections closely resemble smallpox, chickenpox, measles, bacterial skin infections, scabies, medication allergies, and syphilis.^9^ Therefore, early differential diagnosis is crucial to recognizing and restricting the virus's spread to the community. Accurate diagnosis of MPXV is challenging based solely on clinical symptoms, necessitating robust laboratory methods for confirmation. Essential diagnostic procedures encompass viral culture, electron microscopy, and immunohistochemistry for visualizing viral particles. Serology tests detecting anti‐orthopoxvirus immunoglobulin G (IgG) and IgM antibodies provide critical insights. Additionally, conventional PCR and real‐time PCR techniques enable specific genetic identification. The Tetracore Orthopox BioThreat Alert system enhances diagnostic precision. Integration of these molecular assays ensures a comprehensive approach to distinguish MPXV, offering a vital framework for effective case confirmation and epidemiological understanding in outbreaks.^10^

Among all the available options, PCR is the preferred laboratory test due to its higher accuracy and sensitivity.^9^ Additionally, vaccination against smallpox confers about 85% effectiveness in the prevention of monkeypox but vaccine coverage remains insufficient, especially in racial and ethnic minority populations and in low‐ and middle‐income countries, including Pakistan, due to a lack of resources.^11^ Apart from that, the scarcity of diagnostic facilities is an alarming matter requiring urgent attention. Even though most eminent Pakistani laboratories are equipped with PCR machines, they lack the testing kits required. It is imperative that the Pakistani government urgently procures the required testing kits, primers, and reagents to combat a possible viral epidemic.^12^

Given Pakistan's current economic challenges and insufficient healthcare infrastructure, the country is ill‐equipped to manage a new endemic outbreak, especially in the aftermath of COVID‐19.^13^ Therefore, it is imperative to educate the population at risk and train healthcare professionals across the country in the diagnosis and management of the infection. It was observed during the last pandemic that Pakistan was able to control the spread of COVID‐19 better through timely measures and strict control.^13^ Several factors can be attributed to the increased frequency of monkeypox outbreaks over the past four decades; higher population density, increased ease of travel, as well as certain ecological and environmental factors.^2^ The asymptomatic transmission of the infection also contributes to its burden. Therefore, to contain the virus, it is imperative to make testing before flight mandatory and impose travel restrictions on suspected cases. Nongovernmental organizations, civil society organizations and other healthcare entities in Pakistan also played a vital role in monkeypox prevention through surveillance, community education, resource mobilization, and collaboration with authorities, enhancing early detection and healthcare capacity. Henceforth, the adoption of a systemic, coordinated and standardized approach toward such threats is essential. The implantation and adherence to screening can help in early detection and treatment.

In conclusion, the current monkeypox outbreak poses a significant threat to global public health, necessitating a unified response from healthcare providers, government organizations, and the general public. Early detection through rapid diagnostic testing, prompt treatment, and comprehensive prevention strategies are crucial in curbing the transmission of the MPXV and reducing its adverse effects on society. Moreover, sustained research is required to obtain a better understanding of the virus, its transmission pathways and to develop effective therapeutic and prophylactic interventions to prevent future outbreaks.

## AUTHOR CONTRIBUTIONS

**Areeba Fareed**: Conceptualization; methodology; writing—original draft. **Aariz Hussain**: Methodology; writing—original draft. **Fatima Faraz**: Writing—review and editing. **Rima Siblini**: Writing—review and editing.

## CONFLICT OF INTEREST STATEMENT

The authors declare no conflicts of interest.

## TRANSPARENCY STATEMENT

The lead author Rima Siblini affirms that this manuscript is an honest, accurate, and transparent account of the study being reported; that no important aspects of the study have been omitted; and that any discrepancies from the study as planned (and if relevant, registered) have been explained.

## Data Availability

No new data were generated.

## References

[1] Lum FM , Torres‐Ruesta A , Tay MZ , et al. Monkeypox: disease epidemiology, host immunity and clinical interventions. Nat Rev Immunol. 2022;22(10):597‐613. 36064780 10.1038/s41577-022-00775-4 PMC9443635

[2] Prasad S , Galvan Casas C , Strahan AG , et al. A dermatologic assessment of 101 mpox (monkeypox) cases from 13 countries during the 2022 outbreak: skin lesion morphology, clinical course, and scarring. J Am Acad Dermatol. 2023;88(5):1066‐1073. 36641010 10.1016/j.jaad.2022.12.035 PMC9833815

[3] Angelo KM , Smith T , Camprubí‐Ferrer D , et al. Epidemiological and clinical characteristics of patients with monkeypox in the GeoSentinel Network: a cross‐sectional study. Lancet Infect Dis. 2023;23(2):196‐206. 36216018 10.1016/S1473-3099(22)00651-X PMC9546520

[4] World Health Organization . Mpox (monkeypox). WHO [Internet]. April 30, 2023. Accessed April 18, 2023. https://www.who.int/news-room/fact-sheets/detail/monkeypox

[5] World Health Organization . India confirms first case of monkeypox in WHO South‐East Asia Region. WHO [Internet]. April 30, 2023. July 15, 2022. https://www.who.int/southeastasia/news/detail/15-07-2022-india-confirms-first-case-of-monkeypox-in-who-south-east-asia-region

[6] World Health Organization . WHO to assist Pakistan on monkeypox. Digital Association Press of Pakistan. April 30, 2023. Accessed April 28, 2023. https://www.app.com.pk/national/who-to-assist-pakistan-on-monkeypox/

[7] Hussain A . Pakistan confirms its first case of mpox. Al Jazeera [Internet]. April 30, 2023. Accessed April 26, 2023. https://www.aljazeera.com/news/2023/4/26/pakistan-confirms-its-first-case-of-mpox

[8] Bhatti MW . Pakistan detects two monkeypox cases: health ministry. The News International [Internet]. April 30, 2023. Accessed April 25, 2023. https://www.thenews.com.pk/latest/1063910-pakistan-detects-first-monkeypox-case-health-ministry

[9] Saxena SK , Ansari S , Maurya VK , et al. Re‐emerging human monkeypox: a major public‐health debacle. J Med Virol. 2022;95(1):e27902. 35652133 10.1002/jmv.27902

[10] Karagoz A , Tombuloglu H , Alsaeed M , et al. Monkeypox (mpox) virus: classification, origin, transmission, genome organization, antiviral drugs, and molecular diagnosis. J Infect Public Health. 2023;16(4):531‐541. 36801633 10.1016/j.jiph.2023.02.003 PMC9908738

[11] Harapan H , Ophinni Y , Megawati D , et al. Monkeypox: a comprehensive review. Viruses. 2022;14(10):2155. 36298710 10.3390/v14102155 PMC9612348

[12] Najeeb H , Huda Z . Monkeypox virus: a spreading threat for Pakistan? Ann Med Surg. 2022;79:103977. 10.1016/j.amsu.2022.103977 PMC9289342 35860147

[13] Farooq S , Haider SI , Sachwani S , Parpio YN . Insight into COVID‐19 responses and initiatives from Pakistan. J Coll Physicians Surg Pak. 2020;30(6):50‐52. 32723451 10.29271/jcpsp.2020.Supp1.S50

